# Evolutionary Algorithm-Based Crystal Structure Prediction of Cu*_x_*Zn*_y_*O*_z_* Ternary Oxides

**DOI:** 10.3390/molecules28165986

**Published:** 2023-08-10

**Authors:** Mikhail S. Kuklin, Antti J. Karttunen

**Affiliations:** Department of Chemistry and Materials Science, Aalto University, P.O. Box 16100, FI-00076 Espoo, Finland

**Keywords:** crystal structure prediction, evolutionary algorithms, density functional theory, ternary oxides, copper oxides, zinc oxides

## Abstract

Binary zinc(II) oxide (ZnO) and copper(II) oxide (CuO) are used in a number of applications, including optoelectronic and semiconductor applications. However, no crystal structures have been reported for ternary Cu-Zn-O oxides. In that context, we investigated the structural characteristics and thermodynamics of Cu*_x_*Zn*_y_*O*_z_* ternary oxides to map their experimental feasibility. We combined evolutionary crystal structure prediction and quantum chemical methods to investigate potential Cu*_x_*Zn*_y_*O*_z_* ternary oxides. The USPEX algorithm and density functional theory were used to screen over 4000 crystal structures with different stoichiometries. When comparing compositions with non-magnetic Cu^I^ ions, magnetic Cu^II^ ions, and mixed Cu^I^-Cu^II^ compositions, the magnetic Cu_2_Zn_2_O_4_ system is thermodynamically the most favorable. At ambient pressures, the thermodynamically most favorable ternary crystal structure is still 2.8 kJ/mol per atom higher in Gibbs free energy compared to experimentally known binary phases. The results suggest that thermodynamics of the hypothetical Cu*_x_*Zn*_y_*O*_z_* ternary oxides should also be evaluated at high pressures. The predicted ternary materials are indirect band gap semiconductors.

## 1. Introduction

*d*-metal oxides are used in a variety of technological applications such as catalysis [1], optoelectronic applications [2,3], and thermoelectric energy conversion [4]. Recently, many copper-based and iron-based ternary, Earth-abundant oxides have been investigated as photocathode materials in photoelectrochemical cells for water splitting [5]. Considering the widely useful optoelectronic and semiconducting properties of binary ZnO and CuO oxides, one could consider that ternary Cu-Zn-O oxides could possess interesting properties for photoelectrochemical applications. However, ternary Cu*_x_*Zn*_y_*O*_z_* oxides reported to date are typically Cu-doped ZnO or Zn-doped CuO and true ternary phases like CuZnO_2_ are not known. For example, Jin et al. studied the hexagonal-to-cubic phase transition of Zn_0.854_Cu_0.146_O under high pressure, where the material had a ZnO-like wurtzite or rock salt crystal structure [6]. Prabhakaran and Boothroyd studied the single-crystal growth of Zn-doped CuO, Cu_1−*x*_Zn*_x_*O, where *x* was 0.05 or 0.1 and the crystal structure was the monoclinic CuO (tenorite) crystal structure [7]. Analogously, Bououdina et al. studied nanostructured wurtzite-ZnO doped with up to 10% Cu using neutron diffraction and ab initio calculations [8]. The optical properties of such Cu-doped ZnO were also studied with density functional theory (DFT) by Volnianska and Bogusławski [9]. Wattoo et al. investigated the effect of zinc concentration on the physical properties of CuO, where *x* was varied between 0 and 0.08 in Cu_1−*x*_Zn*_x_*O [10]. When Amaral et al. studied the Zn doping effect on the structural properties of CuO, their Cu_1−*x*_Zn*_x_*O samples showed small amounts of spurious phases above *x* = 0.05 [11]. Overall, it appears that Cu*_x_*Zn*_y_*O*_z_* ternary phases have been studied only for compositions where one metal corresponds to about 90% of the total metal content.

While previous experimental studies on ternary Cu-Zn-O oxides have mainly focused on materials where Cu and Zn are in oxidation state +II, Cu often also shows oxidation state +I in various copper oxides. The prototypical example is the copper(I) oxide Cu_2_O. The oxidation state of copper also affects the magnetic properties of the potential ternary oxide: Cu(I) with the 3*d*^10^ valence electron configuration is diamagnetic, while Cu(II) with the 3*d*^9^ valence electron configuration is magnetic. Thus, ternary Cu-Zn-O oxides could in principle exist as non-magnetic Cu(I)-Zn(II)-O compounds, magnetic Cu(II)-Zn(II)-O compounds, or as magnetic, mixed Cu(I)-Cu(II)-Zn(II)-O compounds. Based on the current experimental knowledge of the Cu-Zn-O ternary system, it is not clear which of these choices would be thermodynamically the most favorable one. Given the scarcity of experimental data, the design and synthesis of novel ternary Cu*_x_*Zn*_y_*O*_z_* oxides could be facilitated by a systematic computational investigation of the thermodynamics of hypothetical ternary Cu*_x_*Zn*_y_*O*_z_* oxides. However, thermodynamic studies are hindered by the lack of crystal structures in the hypothetical ternary Cu*_x_*Zn*_y_*O*_z_* oxides. Therefore, the first step in this direction would be to carry out a crystal structure prediction study to shed light on the structural principles of the various non-magnetic and magnetic ternary Cu*_x_*Zn*_y_*O*_z_* oxides.

The starting point in crystal structure prediction is as follows: Given a chemical composition such as Cu_2_Zn_2_O_4_, what is the thermodynamically most stable crystal structure? This global optimization problem should not be considered trivial given the complex and multidimensional energy landscapes of many-atom crystal structures [12]. State-of-the-art quantum chemical methods can very robustly find local minima, but the global optimization of a crystal structure remains a more challenging problem. In the past 20 years, a number of approaches and algorithms has been developed for crystal structure prediction [12]. Some examples of the developed methods are simulated annealing, basin hopping, minima hopping, particle swarm optimization, genetic algorithms, and evolutionary algorithms. The present study is based on evolutionary algorithms, which are among the most powerful crystal structure prediction methods. In both genetic and evolutionary algorithms, a key concept is the population of individuals (crystal structures). Genetic algorithms (GAs) typically use a binary representation for the individuals (“01001…”). Such binary representation of a crystal structure limits the search to a discretized grid within a pre-determined unit cell. In evolutionary algorithms (EAs), the individuals (crystal structures) are represented with real numbers, corresponding to the lattice parameters and atomic coordinates. This choice makes the evolutionary variation operations (heredity, mutations) more difficult to implement compared to genetic algorithms, but at the same time, it greatly increases the efficiency of the global optimization (the search space is continuous, not discrete).

Here, we use evolutionary crystal structure prediction and quantum chemical methods to find potential Cu*_x_*Zn*_y_*O*_z_* ternary oxides. We study the thermodynamics of the predicted ternary phases with respect to experimentally known binary phases. As Cu(II) ions are magnetic, we also investigate the role of magnetism in the hypothetical ternary oxides. The aim of this work is to provide insights into the structural characteristics and thermodynamics of Cu*_x_*Zn*_y_*O*_z_* ternary oxides, providing guidelines for future experimental work on these potential functional *d*-metal oxides.

## 2. Results

To span the configuration space of an unknown crystal structure such as Cu*_x_*Zn*_y_*O*_z_*, different compositions must be considered. For non-magnetic Cu*_x_*Zn*_y_*O*_z_* with Cu(I) and Zn(II) ions, we investigated the following compositions for the primitive cell (number of screened crystal structures in parentheses): Cu_2_ZnO_2_ (~600), Cu_4_Zn_2_O_4_ (~1600), Cu_6_Zn_3_O_6_ (~400), Cu_8_Zn_4_O_8_ (~400). The compositions were chosen by increasing the number of Cu(I) ions stepwise from 2 to 8.

Magnetic compositions for the crystal structure predictions were divided into two groups. The first group represents compositions with only Cu(II) ions and Zn(II) ions: CuZnO_2_ (~200 crystal structures) and Cu_2_Zn_2_O_4_ (~400 crystal structures). In other words, we first studied the simplest 1:1:2 composition and then a doubled composition with two Cu(II) ions in the primitive cell. A similar strategy was used for the second group of magnetic crystal structures with both Cu(I) and Cu(II) ions: simplest composition Cu^II^Cu^I^_2_Zn_2_O_4_ (~400 crystal structures), double composition Cu^II^_2_Cu^I^_4_Zn_4_O_8_ (~200 crystal structures), and finally, the composition Cu^II^_2_Cu^I^_2_Zn_4_O_7_ (~400 crystal structures). The Cu^II^_2_Cu^I^_2_Zn_4_O_7_ composition was included in the screening due to the equal number of Cu and Zn in the crystal structure. In general, the number of formula units used in the USPEX simulations is limited by the available computational resources, and evolutionary searches with two magnetic Cu^II^ ions already proved to be rather demanding with the hybrid DFT-PBE0 method.

Because the studied hypothetical Cu*_x_*Zn*_y_*O*_z_* ternary materials are completely new and there is no information on their magnetic ground states, we had to investigate both ferromagnetic and antiferromagnetic spin settings to find the magnetic ground state of each crystal structure. However, we noted that many of the Cu_2_Zn_2_O_4_ crystal structures that we tried to predict an antiferromagnetic ground state ended up as analogs of ferromagnetic CuZnO_2_ crystal structures with the same magnetic moments, space groups, and relative energies. The magnetic moments of the spin-up and spin-down Cu(II) ions in the predicted antiferromagnetic Cu_2_Zn_2_O_4_ structures were found to be identical to each other. To simplify further simulations with larger unit cells in the case of mixed Cu^I^-Cu^II^ compositions, we carried out the crystal structure predictions only for ferromagnetic configurations.

Relative energies (Δ*E*) of the predicted Cu*_x_*Zn*_y_*O*_z_* crystal structures were estimated by comparing their total energy with the related binary oxides using the following equation:

Δ*E* [kJ mol^−1^] = *E*(Cu*_x_*Zn*_y_*O*_z_*) − *x E*(Cu_2_O/CuO) − *y E*(ZnO) (1)

In particular, in case of non-magnetic Cu_2_ZnO_2_, relative energies per atom were estimated in the following way:

Δ*E* [kJ mol^−1^ per atom] = (*E*(Cu_2_ZnO_2_) − *E*(Cu_2_O) − *E*(ZnO))/5 (2)

In case of magnetic CuZnO_2_, relative energies were estimated in the following way:

Δ*E* [kJ mol^−1^ per atom] = (*E*(CuZnO_2_) − *E*(CuO) − *E*(ZnO))/4 (3)

In case of mixed Cu^II^Cu^I^_2_Zn_2_O_4_, relative energies were estimated in the following way:

Δ*E* [kJ mol^−1^ per atom] = (*E*(Cu^II^Cu^I^_2_Zn_2_O_4_) − *E*(CuO) − *E*(Cu_2_O) − 2 *E*(ZnO))/9 (4)

The relative energies were evaluated with Equations (1)–(4) for all the lowest-energy crystal structures produced by the USPEX simulations. In the detailed discussion here, we focus on crystal structures where Δ*E* was found to be smaller than 10 kJ mol^−1^ per atom. For these crystal structures, we also estimated the Gibbs free energy Δ*G*, where the phonon contributions were evaluated in two different ways: (1) at the Γ point only, and (2) based on phonon supercells. Relative Gibbs free energies Δ*G* were obtained analogously to Δ*E*, using Equations (1)–(4).

**Figure 1:**
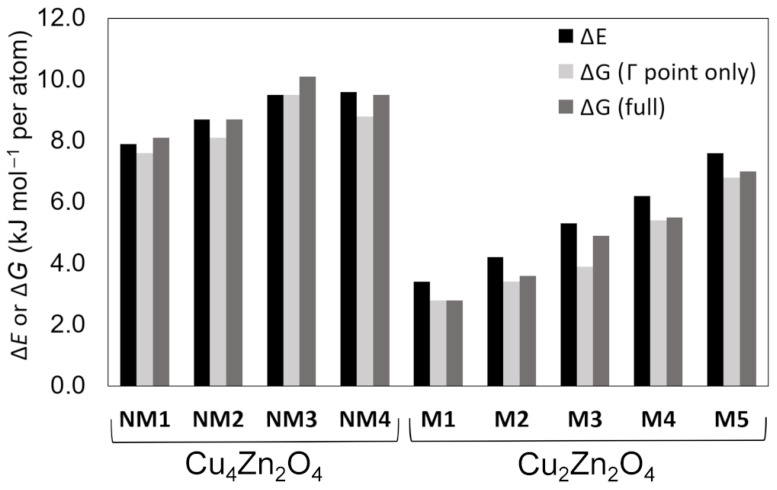
Relative DFT-PBE0-D3/TZVP energies and Gibbs free energies (*T* = 298 K) of the lowest-energy Cu*_x_*Zn*_y_*O*_z_* crystal structures predicted by USPEX. The relative energies are given with respect to Cu_2_O, CuO, and ZnO (see Equations (1)–(4)). Label **NM** refers to non-magnetic crystal structures and label **M** refers to magnetic crystal structures. The number (**NM1**, **NM2**,…) gives the energy ranking based on Δ*E* within this class of crystal structures, **1** being the lowest-energy crystal structure. The relative energies are listed in Table 1.

Figure 1 illustrates the relative total energies and Gibbs free energies of the predicted lowest-energy Cu*_x_*Zn*_y_*O*_z_* crystal structures and Table 1 lists detailed information for them. Optimized lattice parameters are presented in Table 2. The crystal structures are labeled using a scheme that is explained in the caption of Figure 1. The unit cell parameters and atomic coordinates of the predicted lowest-energy structures are given as Supplementary Materials in CIF format.

It seems clear that magnetic Cu*_x_*Zn*_y_*O*_z_* crystal structures possess lower energies than non-magnetic crystal structures. The same is true for Gibbs free energies. None of the mixed crystal structures with both Cu^II^ and Cu^I^ ions were found to have Δ*E* smaller than 10 kJ mol^−1^ per atom. The predicted non-magnetic crystal structures possess a smaller band gap (2.2–2.4 eV) than the magnetic compounds (3.1–3.4 eV).

Coordination numbers of the metal atoms also vary depending on the composition. In the reference binary phases, the coordination numbers of the metal atoms are as follows: Cu in Cu_2_O is two-coordinated (linear), Cu in CuO is four-coordinated (square planar), and Zn in ZnO is four-coordinated (tetrahedral). Non-magnetic crystal structures **NM1**–**NM4** contain linearly coordinated Cu^I^ ions with a coordination number of two, similar to Cu_2_O. In the case of **NM3**, the linear Cu shows clearly bent coordination, with O-Cu-O angles of about 158°. In crystal structures **NM1**–**NM3**, the Zn^II^ ions are four-coordinated with tetrahedral coordination, but the tetrahedrals are somewhat distorted from ideal. In **NM4**, the Zn^II^ atoms are somewhat unexpectedly five-coordinated with square pyramidal coordination. This structure has the highest relative energy of the non-magnetic crystal structures, but its Gibbs free energy is lower compared to **NM3**.

The lowest-energy magnetic crystal structure **M1** has Cu^II^ ions with a coordination number of four and square planar coordination, similar to CuO. Zn^II^ ions in the structure have a coordination number of six, with slightly distorted octahedral coordination. The coordination number of both Cu^II^ and Zn^II^ ions in the magnetic crystal structures **M2**–**M4** is six, with octahedral coordination. Finally, in the magnetic crystal structure **M5**, both Cu^II^ and Zn^II^ ions show a somewhat unexpected five-coordinated square pyramidal coordination sphere.

As mentioned above, we found that the ferromagnetic and antiferromagnetic configurations of magnetic Cu*_x_*Zn*_y_*O*_z_* crystal structures are practically isoenergetic. In fact, ferromagnetic lowest-energy CuZnO_2_ was found to have the same space group and structural parameters as antiferromagnetic **M1**. Energetically, the FM and AFM (**M1**) crystal structures are also very close to each other (for AFM **M1**, Δ*E* = 3.5, Δ*G*^Γ^ = 2.7, Δ*G* = 3.0 kJ mol^–1^ per atom). Thus, to save computational resources, the harmonic frequency calculations of some crystal structures were only carried out with ferromagnetic spin settings. In particular, the antiferromagnetic **M3** structure has space group *P*-1, whereas the ferromagnetic structure possesses space group *C*2/*c*. Antiferromagnetic **M5** has space group *Pm* and its ferromagnetic configuration has space group *Pmn*2_1_. In the case of other **M** crystal structures, no changes in space groups were identified if the magnetic configuration was changed from antiferromagnetic to ferromagnetic.

Δ*E* and Δ*G* estimated at the Γ-point and based on the phonon supercell calculations are in good agreement for the **NM** crystal structures, the maximum difference being 0.8 kJ mol^−1^ per atom. Magnetic **M** crystal structures have similar trends for Δ*E* and Δ*G*. Only in the case of **M3** was the difference between Δ*E* and Δ*G*^Γ^ found to be slightly larger (1.4 kJ mol^−1^ per atom). Δ*G* evaluated at the Γ-point and based on phonon supercell calculations are typically in good agreement with each other. The largest difference is found in the case of crystal structure **M3**, where it is 1.0 kJ mol^−1^ per atom.

**Figure 2:**
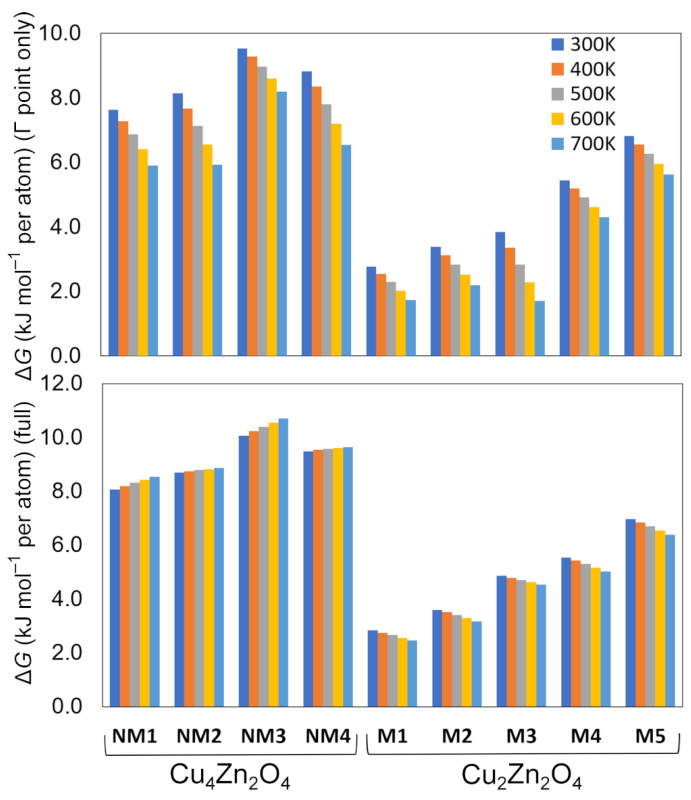
Relative DFT-PBE0-D3/TZVP Gibbs free energies of the lowest-energy Cu*_x_*Zn*_y_*O*_z_* crystal structures at 300–700 K. The (**top**) panel shows Δ*G* evaluated with phonon contributions only at the Γ-point, while the (**bottom**) panel shows Δ*G* from phonon supercell calculations.

We also evaluated the Gibbs free energy for the studied crystal structures at 300, 400, 500, 600, and 700 K (Figure 2). The goal was to see whether the ternary oxides would become more favorable at higher temperatures. Δ*G* evaluated at the Γ-point only decreases for all structures with increasing temperature (top panel in Figure 2). However, when more accurate phonon supercell calculations are considered, the changes are smaller (bottom panel in Figure 2). In fact, for non-magnetic structures, increase in the temperature leads to a small increase in Δ*G*. The largest effect on Δ*G*^Γ^ can be seen for **M3**, where Δ*G*^Γ^ decreases from 3.9 (298 K) to 1.7 kJ mol^−1^ per atom (700 K). However, Δ*G* evaluated from more accurate phonon supercell calculations shows that Δ*G* remains at about ~4 kJ mol^−1^ per atom even at 700 K. Overall, increasing the temperature does not seem to favor the ternary Cu*_x_*Zn*_y_*O*_z_* phases over the experimentally known binary phases Cu_2_O, CuO, and ZnO. However, for the lowest-energy system **M1**, Δ*G* with respect to the binary phases is only 2.8 kJ/mol per atom. This is almost identical to the Gibbs free energy difference between graphite and diamond [14]. Therefore, high-pressure studies on the hypothetical ternary phases should be carried out to see whether higher pressures could favor them over the binary phases.

**Figure 3:**
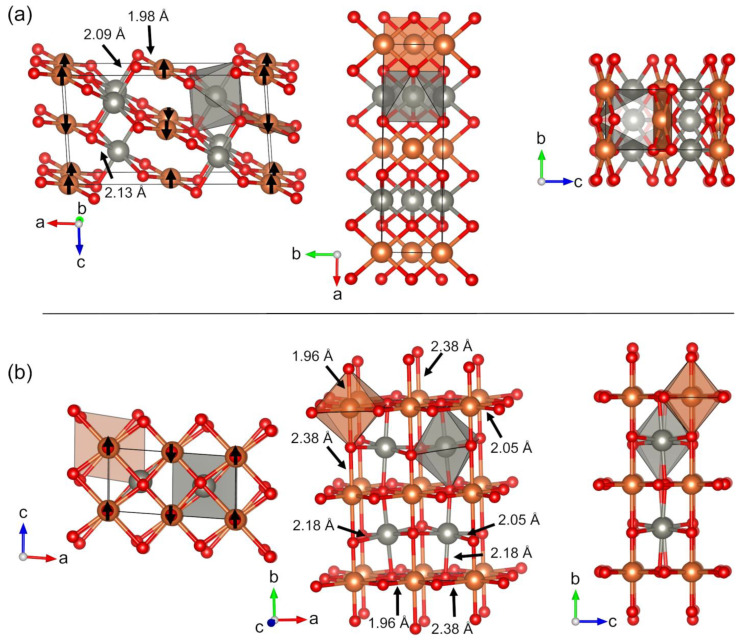
Predicted low-energy crystal structures (**a**) **M1** (*C*2/*m*) and (**b**) **M2** (*C*2/*m*) Cu_2_Zn_2_O_4_ illustrated from three different crystallographic directions. Reddish brown spheres are Cu atoms, gray spheres are Zn atoms, and red spheres are O atoms. Coordination polyhedra are shown for selected Cu and Zn atoms in reddish brown and gray, respectively. The directions of the magnetic moments are illustrated by black arrows.

Magnetic Cu_2_Zn_2_O_4_ crystal structures **M1** and **M2**, illustrated in Figure 3, are the lowest-energy structures predicted here. The monoclinic crystal structure **M1** possesses space group *C*2/*c*. **M1** has the following key interatomic distances: Cu–O distance is 1.98 Å and two Zn–O distances are 2.09 and 2.13 Å. **M1** also has the largest band gap among the studied structures (3.4 eV). Meanwhile, **M2** crystallizes in the monoclinic crystal system with the same *C*2/c space group as **M1**. However, the crystal structure is more complicated compared to **M1,** showing three different Cu–O distances (1.96 Å, 2.05 Å, and 2.38 Å) and two Zn–O distances (2.05 Å and 2.18 Å). The band gap of the **M2** structure is 3.1 eV, which is slightly smaller than that of **M1**. Increasing the temperature does not change the thermodynamic stability order and **M1** remains more favorable than **M2**.

**Figure 4:**
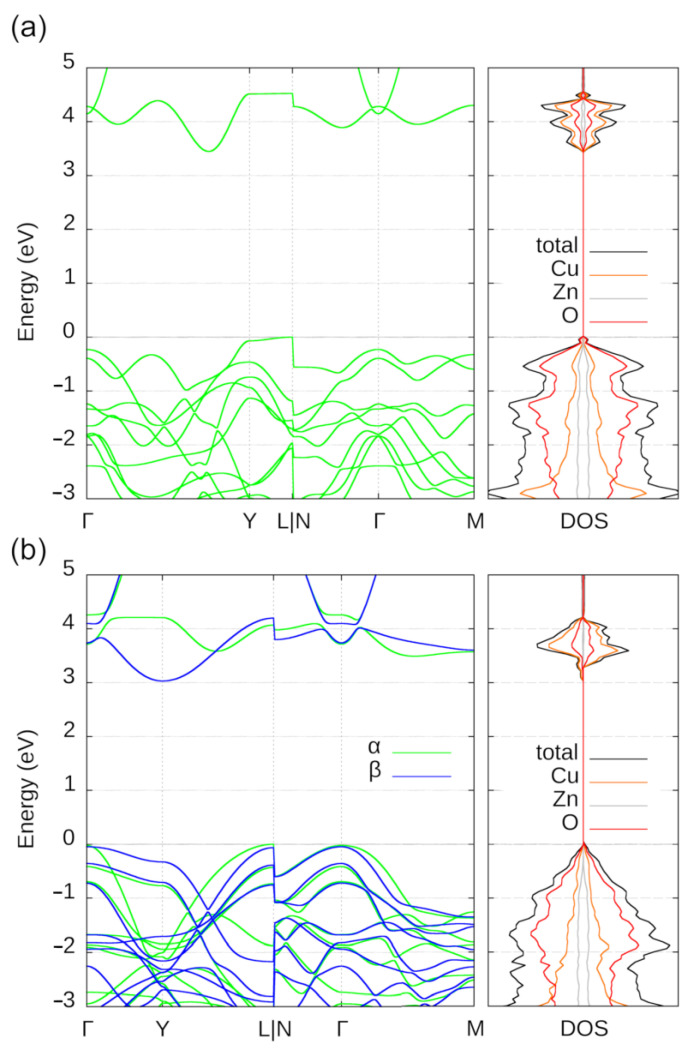
Electronic band structure and density of states of the predicted low-energy Cu_2_Zn_2_O_4_ crystal structures (**a**) **M1** and (**b**) **M2**. For **M1**, the spin-up (α) and spin-down (β) spin channels possess a similar band structure, while for **M2** they are different. The steep step-like changes in the band energies at the L|N connection are simply graphical artefacts due to the plotting software.

All of the low-energy ternary Cu*_x_*Zn*_y_*O*_z_* crystal structures are either semiconductors or wide-band-gap semiconductors based on their predicted DFT-PBE0 band gaps. The electronic band structures and density of states of the lowest-energy crystal structures **M1** and **M2** are illustrated in Figure 4. Both materials possess an indirect band gap (3.4 and 3.1 eV for **M1** and **M2**, respectively). The smallest direct band gap for **M1** is over 4 eV for **M1** and over 3 eV for **M2**. For **M1**, the valence band edge down to −0.2 eV is dominated by oxygen. Below that, Cu contributes more than Zn. The contribution from Zn stays rather similar down to −3 eV, while the contribution from Cu increases and surpasses that of O at around −3 eV. Similarly, in the case of **M2**, Cu contributes more to the topmost valence bands than Zn and the contribution from Zn stays rather similar down to −3 eV, while the contribution from Cu increases and surpasses that of O at around −3 eV. For both materials, the conduction bands are dominated by Cu, with contributions from O and even smaller contributions from Zn (practically negligible in the case of **M2**).

Regarding the magnitude of the predicted band gaps, they are likely to be affected by the known behavior of DFT-PBE0 [15]. For systems where the experimental band gap is smaller than 1 eV, DFT-PBE0 typically significantly overestimates the band gap. For band gaps between 2 and 5 eV, DFT-PBE0 produces more reasonable estimates. For binary Cu_2_O, DFT-PBE0/TZVP yields a band gap of 2.39 eV, while an often-cited experimental result is 2.17 eV [16]. For CuO, DFT-PBE0/TZVP yields a band gap of 3.8 eV, which is clearly overestimated in comparison to experimental 1.7 eV [13]. This behavior should be taken into account when considering the band gaps predicted for the hypothetical ternary oxides here.

## 3. Methods

**Figure 5:**
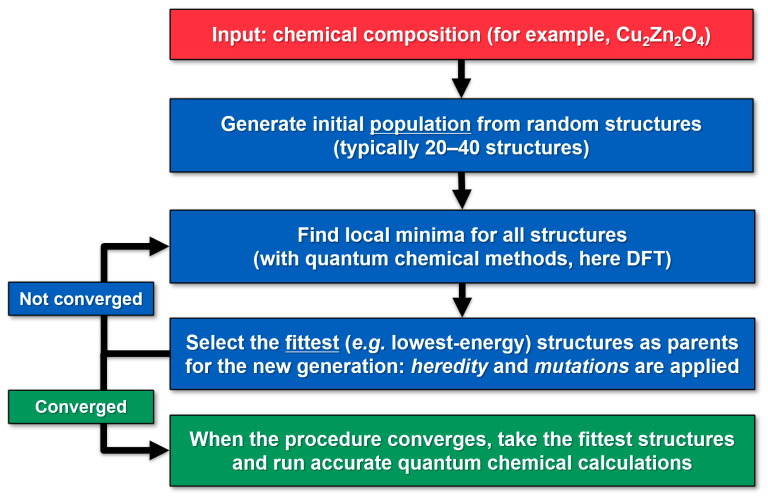
Workflow in USPEX evolutionary crystal structure prediction.

Crystal structure predictions were carried out by using USPEX 9.4.4 code for evolutionary crystal structure prediction [17,18,19]. A typical workflow for a USPEX simulation is presented in Figure 5. The only chemical input for the crystal structure prediction is the composition of the studied system, such as Cu_2_Zn_2_O_4_. In addition, technical parameters related to the USPEX crystal structure prediction algorithm are provided, though these are not highly system-dependent. Similar input files were used for different chemical compositions in this study. An example of the USPEX input file used is given in the Supplementary Materials (Table S1).

The ternary Cu*_x_*Zn*_y_*O*_z_* compositions investigated here are explained in detail in Section 2 “Results”. At the beginning of the simulation, completely random structures are used as a starting point. The space group is chosen randomly, and the Wyckoff positions are filled randomly. Next, local optimizations with quantum chemical methods are carried out for the crystal structures in the starting population and the energies (enthalpies) of the optimized structures are compared with each other. The fittest (lowest-energy) structures are chosen as parents for the new generation, after which new structures are generated by applying heredity and mutations to the parent structures. A typical USPEX run involves thousands of local optimizations, producing hundreds of crystal structures (detailed numbers reported in Section 2 “Results”). The USPEX procedure can be considered converged when the fittest structure is no longer changing after several (e.g., 10) generations. After convergence, the fittest crystal structures still need to be re-optimized at a higher level of theory, as the local optimizations in the USPEX simulations are typically carried out with lower accuracy due to the vast number of calculations.

Quantum chemical calculations within the USPEX simulations were performed using density functional theory (DFT). The CRYSTAL17 and Quantum Espresso (QE) version 6.0 program packages were utilized for the DFT calculations [20,21]. The PBE exchange–correlation functional with GBRV ultrasoft pseudopotentials was used for all DFT calculations carried out with QE [22,23]. It is known that standard generalized gradient approximation (GGA) functionals such as PBE can fail to treat the magnetic moments and electronic structure of systems such as strongly correlated *d*-metal oxides, sometimes even favoring the wrong magnetic ground state [24,25,26,27,28,29,30,31]. Therefore, QE was used only for non-magnetic Cu*_x_*Zn*_y_*O*_z_* compositions, and magnetic Cu*_x_*Zn*_y_*O*_z_* compositions were studied only with CRYSTAL and hybrid density functional methods combined with all-electron basis sets. For CRYSTAL, we used the CRYSTAL interface developed for USPEX, enabling the prediction of magnetic ordering in addition to the crystal structure [32]. The hybrid DFT-PBE0 functional with 25% Hartree–Fock exchange was utilized in CRYSTAL calculations [22,33]. Overall, for a 3*d* metal such as Cu, the use of hybrid DFT over GGA or GGA + *U* is expected to increase the accuracy of the predictions [16,24,34,35,36,37,38]. Therefore, we also carried out USPEX/CRYSTAL simulations for non-magnetic structures to ensure the reliability of the USPEX/QE results. Successful use of the USPEX/CRYSTAL for non-magnetic and magnetic *d*-metal oxides and fluorides has been reported earlier [32,39,40]. All-electron, Gaussian-type split-valence + polarization (SVP) basis sets based on Karlsruhe def2 basis sets were used within the USPEX/CRYSTAL calculations [13,41]. To accelerate the evolutionary searches, the local structure optimizations within USPEX were carried out by using relatively loose convergence criteria. Space group *P*1 was used for the local structure optimizations. The CRYSTAL and QE input files used within the USPEX simulations are given as Supplementary Materials (Tables S2 and S3).

The lowest-energy structures produced by USPEX/CRYSTAL were re-optimized at the DFT-PBE0 level of theory using tighter convergence criteria (“accurate quantum chemical calculations” in Figure 5). Structural optimizations were carried out in the space groups found with the FINDSYM program package [42]. All-electron, Gaussian-type triple-*ζ*-valence + polarization (TZVP) basis sets based on the Karlsruhe def2 basis set were used within the crystal structure predictions [13,41]. Cu(I) ions are expected to show weak “cuprophilic” *d*^10^–*d*^10^ interactions [43,44,45,46], which were taken into account using Grimme’s D3 dispersion correction with zero-damping (ZD) [47,48]. For the re-optimization, the reciprocal space *k*-point meshes were chosen depending on the magnitude of the corresponding direct space lattice parameter *d*: *d* < 4 Å → 12 *k*-points along *d*; 4 Å < *d* < 6 Å → 8 *k*-points, 6 Å < *d* < 8 Å → 6 *k*-points; 8 Å < *d* < 12 Å → 4 *k*-points; *d* > 12 Å → 2 *k*-points. Tightened tolerance factors (TOLINTEG) of 8, 8, 8, 8, and 16 were used for the evaluation of the Coulomb and exchange integrals. All reported structures were confirmed to be true local minima by means of a harmonic frequency calculation [49,50]. Calculations of phonon dispersions were carried out for supercells where *a*, *b*, and *c* lattice parameters were about 10 Å. The optimized structures of the lowest-energy ternary Cu*_x_*Zn*_y_*O*_z_* crystal structures are included as Supplementary Materials in CIF format.

For estimating the energetics and thermodynamics of the predicted ternary Cu*_x_*Zn*_y_*O*_z_* crystal structures, we also optimized the structures of binary Cu_2_O, CuO, and ZnO at the DFT-PBE0-D3(ZD)/TZVP level of theory. All relative energies reported in this paper have been obtained at this level of theory. CuO was studied using an antiferromagnetic ground state [51,52,53,54,55]. Monkhorst–Pack-type 8 × 8 × 8, 4 × 8 × 4, and 12 × 12 × 8 *k*-point meshes were used for Cu_2_O, CuO, and ZnO, respectively. Including the D3(ZD) dispersion correction does not change the lattice parameters of the binary oxides significantly in comparison to DFT-PBE0 without dispersion correction [13]. For Cu_2_O, the optimized lattice parameter *a* is 4.28 Å with D3 and 4.32 Å without D3. For ZnO, the optimized lattice parameters are 3.25 Å and 5.19 Å with D3 and 3.27 Å and 5.21 Å without D3.

## 4. Conclusions

We combined evolutionary crystal structure prediction and quantum chemical methods to discover potential Cu*_x_*Zn*_y_*O*_z_* ternary oxides. Over 4000 crystal structures with different stoichiometries were screened in the ternary system. When comparing compositions with non-magnetic Cu^I^ ions, magnetic Cu^II^ ions, and mixed Cu^I^-Cu^II^ compositions, the magnetic Cu_2_Zn_2_O_4_ system is thermodynamically the most favorable. At ambient pressures, the thermodynamically most favorable crystal structure (**M1**) is still 2.8 kJ/mol per atom higher in Gibbs free energy compared to experimentally known binary phases. However, this energy difference is almost identical to the Gibbs free energy difference between graphite and diamond [14], suggesting that thermodynamics of the hypothetical ternary systems studied here should also be evaluated at high pressures.

## Figures and Tables

**Table 1:** Space group, relative energy Δ*E*, relative Gibbs free energy Δ*G*, band gap, Cu^II^ magnetic moment, and coordination numbers for the lowest-energy Cu*_x_*Zn*_y_*O*_z_* crystal structures predicted in this study. The crystal structures are labeled using a scheme that is explained in the caption of Figure 1.

Crystal Structure		Space Group	Δ*E* (kJ mol^−1^ per atom)	Δ*G*^Γ^ (kJ mol^−1^ per atom) ^b^	Δ*G* (kJ mol^−1^ per atom) ^c^	Band Gap (eV)	Cu^II^ Magnetic Moment (μ_B_)	Coordination Number of Cu	Coordination Number of Zn
Cu_4_Zn_2_O_4_	**NM1**	*Pc* (7)	7.9	7.6	8.1	2.4	–	2	4
	**NM2**	*I*2_1_2_1_2_1_ (24)	8.7	8.1	8.7	2.2	–	2	4
	**NM3**	*Fdd*2(43)	9.5	9.5	10.1	2.4	–	2	4
	**NM4**	*P*-1 (2)	9.6	8.8	9.5	2.2	–	2	5
Cu_2_Zn_2_O_4_	**M1**	*C*2/*m* (12)	3.4	2.8	2.8	3.4	0.7	4	6
	**M2**	*C*2/*m* (12)	4.2	3.4	3.6	3.1	0.7	6	6
	**M3**	*P*-1 (2)/*C*2/*c* (15) ^a^	5.3	3.9	4.9	3.4	0.7	6	6
	**M4**	*P*1 (1)	6.2	5.4	5.5	3.3	0.7	6	6
	**M5**	*Pm* (6)/*Pmn*2_1_ (31) ^a^	7.6	6.8	7.0	3.1	0.7	5	5

^a^ Antiferromagnetic crystal structure, where higher-symmetry ferromagnetic space group was used for harmonic frequency calculations (see text for more details). ^b^ Γ point phonon contributions only. ^c^ Phonon supercell calculation.

**Table 2:** Optimized lattice parameters of the lowest-energy Cu*_x_*Zn*_y_*O*_z_* crystal structures predicted in this study. The crystal structures are labeled using a scheme that is explained in the caption of Figure 1. The lattice parameters of the experimentally known binary oxides are provided, as well ^a^.

Crystal Structure		*a* (Å)	*b* (Å)	*c* (Å)	α (°)	β (°)	γ (°)
Cu_4_Zn_2_O_4_	**NM1**	3.34	7.73	5.74	90	125.1	90
	**NM2**	3.14	9.88	8.06	90	90	90
	**NM3**	7.94	7.64	8.08	90	90	90
	**NM4**	3.05	6.26	6.49	73.6	79.1	82.2
Cu_2_Zn_2_O_4_	**M1**	9.91	2.87	5.46	90	93.1	90
	**M2**	5.98	8.67	2.95	90	93.6	90
	**M3**	2.95	2.95	10.01	98.0	96.1	117.3
	**M4**	2.95	5.13	5.28	97.6	92.7	103.7
	**M5**	4.98	2.84	5.67	90	90.0	90

^a^ Experimental lattice parameters for binary oxides [13]. Cu_2_O: *a* = 4.267 Å; *a* = CuO: 4.6797 Å, *b* = 3.4314 Å, *c* = 5.1362 Å, β = 99.262°; ZnO: *a* = 3.24986 Å; *c* = 5.20662 Å.

## Data Availability

Not applicable.

## Supplementary Materials

The following supporting information can be downloaded at: https://www.mdpi.com/article/10.3390/molecules28165986/s1, Table S1: USPEX input file for Cu*_x_*Zn*_y_*O*_z_* crystal structure prediction; Table S2: Quantum Espresso input files used in USPEX simulations; Table S3: CRYSTAL input files used in USPEX simulations. Crystal structure data are included in CIF format.

## References

[1] Védrine J.C. (2019). Metal Oxides in Heterogeneous Oxidation Catalysis: State of the Art and Challenges for a More Sustainable World. ChemSusChem.

[2] Dixon S.C., Scanlon D.O., Carmalt C.J., Parkin I.P. (2016). N-Type Doped Transparent Conducting Binary Oxides: An Overview. J. Mater. Chem. C.

[3] Spencer J.A., Mock A.L., Jacobs A.G., Schubert M., Zhang Y., Tadjer M.J. (2022). A Review of Band Structure and Material Properties of Transparent Conducting and Semiconducting Oxides: Ga_2_O_3_, Al_2_O_3_, In_2_O_3_, ZnO, SnO_2_, CdO, NiO, CuO, and Sc_2_O_3_. Appl. Phys. Rev..

[4] Walia S., Balendhran S., Nili H., Zhuiykov S., Rosengarten G., Wang Q.H., Bhaskaran M., Sriram S., Strano M.S., Kalantar-zadeh K. (2013). Transition Metal Oxides—Thermoelectric Properties. Prog. Mater. Sci..

[5] Díez-García M.I., Gómez R. (2022). Progress in Ternary Metal Oxides as Photocathodes for Water Splitting Cells: Optimization Strategies. Sol. RRL.

[6] Jin Y., Zhang J., Zhu P., Gao W., Cui Q. (2011). The Phase Transition of Zn0.854Cu0.146O under High Pressure. Phys. Status Solidi B.

[7] Prabhakaran D., Boothroyd A.T. (2003). Single Crystal Growth of Zn-Doped CuO by the Floating-Zone Method. J. Cryst. Growth.

[8] Bououdina M., Mamouni N., Lemine O.M., Al-Saie A., Jaafar A., Ouladdiaf B., El Kenz A., Benyoussef A., Hlil E.K. (2012). Neutron Diffraction Study and Ab-Initio Calculations of Nanostructured Doped ZnO. J. Alloys Compd..

[9] Volnianska O., Bogusławski P. (2019). Green Luminescence and Calculated Optical Properties of Cu Ions in ZnO. J. Alloys Compd..

[10] Wattoo A.G., Song Z., Iqbal M.Z., Rizwan M., Saeed A., Ahmad S., Ali A., Naz N.A. (2015). Effect of Zinc Concentration on Physical Properties of Copper Oxide (Cu1−xZnxO). J. Mater. Sci. Mater. Electron..

[11] Amaral J.B., Araujo R.M., Pedra P.P., Meneses C.T., Duque J.G.S., Rezende M.D.S. (2016). Doping Effect on the Structural Properties of Cu_1−*x*_(Ni, Zn, Al and Fe)*_x_*O Samples (0 < *x* < 0.10): An Experimental and Computational Study. J. Solid State Chem..

[12] Oganov A.R., Pickard C.J., Zhu Q., Needs R.J. (2019). Structure Prediction Drives Materials Discovery. Nat. Rev. Mater..

[13] Kuklin M.S., Eklund K., Linnera J., Ropponen A., Tolvanen N., Karttunen A.J. (2022). Structural Properties and Magnetic Ground States of 100 Binary D-Metal Oxides Studied by Hybrid Density Functional Methods. Molecules.

[14] Popov I.V., Görne A.L., Tchougréeff A.L., Dronskowski R. (2019). Relative Stability of Diamond and Graphite as Seen through Bonds and Hybridizations. Phys. Chem. Chem. Phys..

[15] Civalleri B., Presti D., Dovesi R., Savin A., Springborg M. (2012). On Choosing the Best Density Functional Approximation. Chemical Modelling.

[16] Linnera J., Karttunen A.J. (2017). Ab Initio Study of the Lattice Thermal Conductivity of Cu2O Using the Generalized Gradient Approximation and Hybrid Density Functional Methods. Phys. Rev. B.

[17] Glass C.W., Oganov A.R., Hansen N. (2006). USPEX—Evolutionary Crystal Structure Prediction. Comput. Phys. Commun..

[18] Oganov A.R., Lyakhov A.O., Valle M. (2011). How Evolutionary Crystal Structure Prediction Works—And Why. Acc. Chem. Res..

[19] Lyakhov A.O., Oganov A.R., Stokes H.T., Zhu Q. (2013). New Developments in Evolutionary Structure Prediction Algorithm USPEX. Comput. Phys. Commun..

[20] Dovesi R., Erba A., Orlando R., Zicovich-Wilson C.M., Civalleri B., Maschio L., Rérat M., Casassa S., Baima J., Salustro S. (2018). Quantum-Mechanical Condensed Matter Simulations with CRYSTAL. Wiley Interdiscip. Rev. Comput. Mol. Sci..

[21] Giannozzi P., Baroni S., Bonini N., Calandra M., Car R., Cavazzoni C., Ceresoli D., Chiarotti G.L., Cococcioni M., Dabo I. (2009). QUANTUM ESPRESSO: A Modular and Open-Source Software Project for Quantum Simulations of Materials. J. Phys. Condens. Matter.

[22] Perdew J.P., Burke K., Ernzerhof M. (1996). Generalized Gradient Approximation Made Simple. Phys. Rev. Lett..

[23] Garrity K.F., Bennett J.W., Rabe K.M., Vanderbilt D. (2014). Pseudopotentials for High-Throughput DFT Calculations. Comput. Mater. Sci..

[24] Wang L., Maxisch T., Ceder G. (2006). Oxidation Energies of Transition Metal Oxides within the GGA+U Framework. Phys. Rev. B.

[25] Schrön A., Rödl C., Bechstedt F. (2012). Crystalline and Magnetic Anisotropy of the 3d-Transition Metal Monoxides MnO, FeO, CoO, and NiO. Phys. Rev. B.

[26] Noh J., Osman O.I., Aziz S.G., Winget P., Brédas J.L. (2014). A Density Functional Theory Investigation of the Electronic Structure and Spin Moments of Magnetite. Sci. Technol. Adv. Mater..

[27] Lima A.F. (2016). Density Functional Theory Study on the Magnetic Properties of Co3O4with Normal Spinel Structure. J. Phys. Chem. Solids.

[28] Singh V., Kosa M., Majhi K., Major D.T. (2015). Putting DFT to the Test: A First-Principles Study of Electronic, Magnetic, and Optical Properties of Co3O4. J. Chem. Theory Comput..

[29] Deng H.X., Li J., Li S.S., Xia J.B., Walsh A., Wei S.H. (2010). Origin of Antiferromagnetism in CoO: A Density Functional Theory Study. Appl. Phys. Lett..

[30] Bredow T., Gerson A.R. (2000). Effect of Exchange and Correlation on Bulk Properties of MgO, NiO, and CoO. Phys. Rev. B.

[31] Rollmann G., Rohrbach A., Entel P., Hafner J. (2004). First-Principles Calculation of the Structure and Magnetic Phases of Hematite. Phys. Rev. B.

[32] Kuklin M.S., Karttunen A.J. (2018). Crystal Structure Prediction of Magnetic Transition Metal Oxides by Using Evolutionary Algorithm and Hybrid DFT Methods. J. Phys. Chem. C.

[33] Adamo C., Barone V. (1999). Toward Reliable Density Functional Methods without Adjustable Parameters: The PBE0 Model. J. Chem. Phys..

[34] Rödl C., Fuchs F., Furthmüller J., Bechstedt F. (2009). Quasiparticle Band Structures of the Antiferromagnetic Transition-Metal Oxides MnO, FeO, CoO, and NiO. Phys. Rev. B.

[35] Kulik H.J., Marzari N. (2011). Transition-Metal Dioxides: A Case for the Intersite Term in Hubbard-Model Functionals. J. Chem. Phys..

[36] Chen X., Parker D., Du M.H., Singh D.J. (2013). Potential Thermoelectric Performance of Hole-Doped Cu2O. New J. Phys..

[37] Seo D.H., Urban A., Ceder G. (2015). Calibrating Transition-Metal Energy Levels and Oxygen Bands in First-Principles Calculations: Accurate Prediction of Redox Potentials and Charge Transfer in Lithium Transition-Metal Oxides. Phys. Rev. B.

[38] Gillen R., Robertson J. (2013). Accurate Screened Exchange Band Structures for the Transition Metal Monoxides MnO, FeO, CoO and NiO. J. Phys. Condens. Matter.

[39] Kuklin M.S., Maschio L., Usvyat D., Kraus F., Karttunen A.J. (2019). Evolutionary Algorithm-Based Crystal Structure Prediction for Copper(I) Fluoride. Chem. Eur. J..

[40] Eklund K., Kuklin M.S., Kraus F., Karttunen A.J. (2020). Evolutionary Algorithm-based Crystal Structure Prediction for Gold(I) Fluoride. ChemPhysChem.

[41] Weigend F., Ahlrichs R. (2005). Balanced Basis Sets of Split Valence, Triple Zeta Valence and Quadruple Zeta Valence Quality for H to Rn: Design and Assessment of Accuracy. Phys. Chem. Chem. Phys..

[42] Stokes H.T., Hatch D.M. (2005). *FINDSYM*: Program for Identifying the Space-Group Symmetry of a Crystal. J. Appl. Crystallogr..

[43] Pyykko P., Runeberg N., Mendizabal F. (1997). Theory of the D10-D10 Closed-Shell Attraction: 1. Dimers Near Equilibrium. Chem. Soc. Rev..

[44] Hermann H.L., Boche G., Schwerdtfeger P. (2001). Metallophilic Interactions in Closed-Shell Copper (I) Compounds—A Theoretical Study. Chem Eur J.

[45] Grady E.O., Kaltsoyannis N. (2004). Does Metallophilicity Increase or Decrease down Group 11? Computational Investigations of [Cl–M–PH_3_]_2_ (M = Cu, Ag, Au, [111]). Phys Chem Chem Phys.

[46] Angels Carvajal M., Alvarez S., Novoa J.J. (2004). The Nature of Intermolecular CuI...CuI Interactions: A Combined Theoretical and Structural Database Analysis. Chem Eur J.

[47] Grimme S., Antony J., Ehrlich S., Krieg H. (2010). A Consistent and Accurate Ab Initio Parametrization of Density Functional Dispersion Correction (DFT-D) for the 94 Elements H-Pu. J. Chem. Phys..

[48] Grimme S., Hansen A., Brandenburg J.G., Bannwarth C. (2016). Dispersion-Corrected Mean-Field Electronic Structure Methods. Chem. Rev..

[49] Zicovich-Wilson C.M., Pascale F., Roetti C., Saunders V.R., Orlando R., Dovesi R. (2004). Calculation of the Vibration Frequencies of A-Quartz: The Effect of Hamiltonian and Basis Set. J. Comput. Chem..

[50] Pascale F., Zicovich-Wilson C.M., Lopez Gejo F., Civalleri B., Orlando R., Dovesi R. (2004). The Calculation of the Vibrational Frequencies of Crystalline Compounds and Its Implementation in the CRYSTAL Code. J. Comput. Chem..

[51] Åsbrink S., Norrby L.J. (1970). A Refinement of the Crystal Structure of Copper(II) Oxide with a Discussion of Some Exceptional e.s.d.’s. Acta Crystallogr. B.

[52] Forsyth J.B., Brown P.J., Wanklyn B.M. (1988). Magnetism in Cupric Oxide. J. Phys. C Solid State Phys..

[53] Yang B., Tranquada J., Shirane G. (1988). Neutron Scattering Studies of the Magnetic Structure of Cupric Oxide. Phys. Rev. B Condens. Matter Mater. Phys..

[54] Yang B.X., Thurston T.R., Tranquada J.M., Shirane G. (1989). Magnetic Neutron Scattering Study of Single-Crystal Cupric Oxide. Phys. Rev. B.

[55] Rödl C., Sottile F., Reining L. (2015). Quasiparticle Excitations in the Photoemission Spectrum of CuO from First Principles: A GW Study. Phys. Rev. B Condens. Matter Mater. Phys..

