# Supplementary material for: Evolutionary Algorithm-Based Crystal Structure Prediction of CuxZnyOz Ternary Oxides
Source: Molecules. 2023 Aug 10;28(16):5986. doi: 10.3390/molecules28165986 (PMC10459973; doi:10.3390/molecules28165986)
Supplement: Supplementary file 1 [file molecules-28-05986-s001.zip › molecules-2523903-supplementary.pdf]

Supplementary Materials for

Evolutionary Algorithm -based Crystal Structure Prediction of $Cu_xZn_yO_z$ Ternary Oxides

Mikhail S. Kuklin and Antti J. Karttunen *

Department of Chemistry and Materials Science, Aalto University, P.O. Box 16100, FI-00076 Espoo, Finland
* Correspondence: antti.karttunen@aalto.fi

Contents

Table S1. USPEX input file for $Cu_xZn_yO_z$ crystal structure prediction.
Table S2. Quantum Espresso input files used in USPEX simulations.
Table S3. CRYSTAL input files used in USPEX simulations.

Table S1. USPEX input files for $Cu_xZn_yO_z$ crystal structure prediction.

USPEX/Quantum Espresso input file for composition Cu_2ZnO_2:

```
******************************************
*      TYPE OF RUN AND SYSTEM            *
******************************************

USPEX : calculationMethod (USPEX, VCNEB, META)
300   : calculationType (dimension: 0-3; molecule: 0/1; varcomp: 0/1)
1     : optType (optimise by: 1=enthalpy, 2=volume, 3=hardness,...)
1     : AutoFrac

% atomType
Cu Zn O
% EndAtomType

% numSpecies
2 1 2
% EndNumSpecies

% symmetries
2-230
% EndSymmetries

******************************************
*               POPULATION               *
******************************************
20    : populationSize
30    : numGenerations
10    : stopCrit
0     : reoptOld
******************************************
*          VARIATION OPERATORS           *
******************************************
0.50  : fracGene
0.20  : fracRand
0.10  : fracAtomsMut
0.10  : fracLatMut
0.10  : fracPerm

*****************************************
*   DETAILS OF AB INITIO CALCULATIONS   *
*****************************************
% abinitioCode
8 8 8
% EndAbinit

% KresolStart
0.14 0.12 0.08
% Kresolend
```

USPEX/CRYSTAL input file for composition $Cu_2Zn_2O_4$:

```
*****************************************
*      TYPE OF RUN AND SYSTEM           *
*****************************************
USPEX : calculationMethod (USPEX, VCNEB, META)
300   : calculationType (dimension: 0-3; molecule: 0/1; varcomp: 0/1)
1     : optType (optimise by: 1=enthalpy, 2=volume, 3=hardness,...)
1     : AutoFrac

% atomType
Cu Zn O
% EndAtomType

% numSpecies
2 2 4
% EndNumSpecies

% symmetries
2-230
% EndSymmetries

*****************************************
*               POPULATION              *
*****************************************
20     : populationSize
30    : numGenerations
10    : stopCrit
0     : reoptOld
*****************************************
*          VARIATION OPERATORS          *
*****************************************
0.50  : fracGene
0.20  : fracRand
0.10  : fracAtomsMut
0.10  : fracLatMut
0.10  : fracPerm
****************************************
*             CONSTRAINTS              *
****************************************
% IonDistances
1.7 1.7 1.3
1.7 1.8 1.4
1.3 1.4 1.0
% EndDistances

****************************************
*   DETAILS OF AB INITIO CALCULATIONS   *
****************************************
% abinitioCode
20 20 20
% EndAbinit

% KresolStart
0.14 0.12 0.08
% Kresolend


0 : doSpaceGroup
```

USPEX input files for other $Cu_xZn_yO_z$ compositions are similar but with different number of atoms (*numSpecies* keyword).

Table S2. Quantum Espresso input files used within USPEX simulations.

First relaxation step:

```
&CONTROL
  calculation  = 'vc-relax'
  restart_mode = 'from_scratch'
  prefix       = 'cu2zno2'
  wf_collect   = .false.
  disk_io = 'none'
  etot_conv_thr=1.0D-3
  forc_conv_thr=1.0D-2
  nstep = 25
/
&SYSTEM
  ibrav     = 0
  nat       = AAAA
  ntyp      = BBBB
  ecutwfc   = 40
  ecutrho   = 200
  nosym     = .true.
  occupations = 'smearing'
  smearing = 'gaussian'
  degauss = 0.02
/
&ELECTRONS
  conv_thr = 5.D-04
  mixing_beta = 0.7
  electron_maxstep = 100
/
&IONS
/
&CELL
  cell_factor = 2.0
/
ATOMIC_SPECIES
 Cu 63.546 cu_pbe_v1.2.uspp.F.UPF
 Zn 65.38 zn_pbe_v1.uspp.F.UPF
 O 15.999 o_pbe_v1.2.uspp.F.UPF
```

Second relaxation step:

```
&CONTROL
  calculation  = 'vc-relax'
  restart_mode = 'from_scratch'
  prefix       = 'cu2zno2'
  wf_collect   = .false.
  disk_io = 'none'
  etot_conv_thr=1.0D-3
  forc_conv_thr=1.0D-2
  nstep = 25
/
&SYSTEM
  ibrav     = 0
  nat       = AAAA
  ntyp      = BBBB
  ecutwfc   = 40
  ecutrho   = 200
  nosym     = .true.
  occupations = 'smearing'
  smearing = 'gaussian'
  degauss = 0.02
/
&ELECTRONS
  conv_thr = 1.D-05
  mixing_beta = 0.7
  electron_maxstep = 100
/
&IONS
/
&CELL
  cell_factor = 2.0
/
ATOMIC_SPECIES
 Cu 63.546 cu_pbe_v1.2.uspp.F.UPF
 Zn 65.38 zn_pbe_v1.uspp.F.UPF
 O 15.999 o_pbe_v1.2.uspp.F.UPF
```

Third relaxation step:

```
&CONTROL
  calculation  = 'vc-relax'
  restart_mode = 'from_scratch'
  prefix       = 'cu2zno2'
  wf_collect   = .false.
  disk_io = 'none'
  etot_conv_thr=1.0D-4
  forc_conv_thr=1.0D-3
  nstep = 30
/
&SYSTEM
  ibrav     = 0
  nat       = AAAA
  ntyp      = BBBB
  ecutwfc   = 40
  ecutrho   = 200
  nosym     = .true.
  occupations = 'smearing'
  smearing = 'gaussian'
  degauss = 0.02
/
&ELECTRONS
  conv_thr = 1.D-06
  mixing_beta = 0.7
  electron_maxstep = 100
/
&IONS
/
&CELL
  cell_factor = 2.0
/
ATOMIC_SPECIES
 Cu 63.546 cu_pbe_v1.2.uspp.F.UPF
 Zn 65.38 zn_pbe_v1.uspp.F.UPF
 O 15.999 o_pbe_v1.2.uspp.F.UPF
```

Table S3. CRYSTAL input files used within USPEX simulations

Basis set section is omitted. Some of the USPEX simulations were implemented with TOLINTEG 8, 8, 8, 8 and 16 for the third relaxation step. Some minor changes in the input files (for example, *FMIXING*) are also possible depending on the studied composition.

First relaxation step:

```
###
EXTERNAL
OPTGEOM
MAXCYCLE
30
TOLDEE
4
TOLDEG
0.03
TOLDEX
0.12
ENDOPT
END
#Basis set
99 0
END
DFT
PBE0
LGRID
SPIN
END
TOLINTEG
7 7 7 7 14
TOLDEE
5
PPAN
ATOMSPIN
2
1 1 2 -1
FMIXING
80
SMEAR
0.005
MAXCYCLE
30
EXCHSIZE
30000000
BIPOSIZE
30000000
#Shrinking factor
```

Second relaxation step:

```
###
EXTERNAL
OPTGEOM
MAXCYCLE
30
TOLDEE
4
TOLDEG
0.0075
TOLDEX
0.03
ENDOPT
END
#Basis set
99 0
END
DFT
PBE0
LGRID
SPIN
END
TOLINTEG
7 7 7 7 14
TOLDEE
5
PPAN
ATOMSPIN
2
1 1 2 -1
FMIXING
80
SMEAR
0.002
MAXCYCLE
50
EXCHSIZE
30000000
BIPOSIZE
30000000
#Shrinking factor
```

Third relaxation step:

```
###
EXTERNAL
OPTGEOM
MAXCYCLE
50
TOLDEE
5
TOLDEG
0.0015
TOLDEX
0.006
ENDOPT
END
#Basis set
99 0
END
DFT
PBE0
LGRID
SPIN
END
TOLINTEG
7 7 7 7 14
TOLDEE
6
PPAN
ATOMSPIN
2
1 1 2 -1
FMIXING
80
MAXCYCLE
50
EXCHSIZE
30000000
BIPOSIZE
30000000
#Shrinking factor
```
